# Decoding Radiomics: A Step-by-Step Guide to Machine Learning Workflow in Hand-Crafted and Deep Learning Radiomics Studies

**DOI:** 10.3390/diagnostics14222473

**Published:** 2024-11-05

**Authors:** Maurizio Cè, Marius Dumitru Chiriac, Andrea Cozzi, Laura Macrì, Francesca Lucrezia Rabaiotti, Giovanni Irmici, Deborah Fazzini, Gianpaolo Carrafiello, Michaela Cellina

**Affiliations:** 1 Postgraduation School in Radiodiagnostics, Università degli Studi di Milano, Via Festa del Perdono 7, 20122 Milan, Italy; maurizio.ce@unimi.it (M.C.); laura.macri@unimi.it (L.M.); francesca.rabaiotti@unimi.it (F.L.R.); 2 Politecnico di Milano, Piazza Leonardo da Vinci 32, 20133 Milan, Italy; redarked@gmail.com; 3 Imaging Institute of Southern Switzerland (IIMSI), Ente Ospedaliero Cantonale (EOC), Via Tesserete 46, 6900 Lugano, Switzerland; andrea.cozzi@gmail.com; 4 Breast Imaging Department, Fondazione IRCCS Istituto Nazionale dei Tumori, Via Giacomo Venezian 1, 20133 Milan, Italy; irmici.giovanni25@gmail.com; 5 Radiology Department, Centro Diagnostico Italiano, Via Saint Bon 20, 20147 Milan, Italy; deborah.fazzini@cdi.it; 6 Radiology Department, Fondazione IRCCS Cà Granda Ospedale Maggiore Policlinico, Via Francesco Sforza 35, 20122 Milan, Italy; gcarraf@gmail.com; 7 Department of Oncology and Hematology-Oncology, Università degli Studi di Milano, Via Festa del Perdono 7, 20122 Milan, Italy; 8 Radiology Department, ASST Fatebenefratelli Sacco, Piazza Principessa Clotilde 3, 20121 Milan, Italy

**Keywords:** radiomics, radiomics pipeline, machine learning, deep learning, METRICS, AI

## Abstract

Although radiomics research has experienced rapid growth in recent years, with numerous studies dedicated to the automated extraction of diagnostic and prognostic information from various imaging modalities, such as CT, PET, and MRI, only a small fraction of these findings has successfully transitioned into clinical practice. This gap is primarily due to the significant methodological challenges involved in radiomics research, which emphasize the need for a rigorous evaluation of study quality. While many technical aspects may lie outside the expertise of most radiologists, having a foundational knowledge is essential for evaluating the quality of radiomics workflows and contributing, together with data scientists, to the development of models with a real-world clinical impact. This review is designed for the new generation of radiologists, who may not have specialized training in machine learning or radiomics, but will inevitably play a role in this evolving field. The paper has two primary objectives: first, to provide a clear, systematic guide to radiomics study pipeline, including study design, image preprocessing, feature selection, model training and validation, and performance evaluation. Furthermore, given the critical importance of evaluating the robustness of radiomics studies, this review offers a step-by-step guide to the application of the METhodological RadiomICs Score (METRICS, 2024)—a newly proposed tool for assessing the quality of radiomics studies. This roadmap aims to support researchers and reviewers alike, regardless of their machine learning expertise, in utilizing this tool for effective study evaluation.

## 1. Introduction

In recent years, radiomics has emerged as a prominent topic in medical literature, highlighting the growing importance of artificial intelligence applications in medicine, particularly in radiology [1,2,3]. Radiomics refers to the extraction and analysis of large sets of quantitative features from medical images [4,5]. From now on, unless otherwise specified, we will use the term “radiomics” in its broadest sense, including all AI-driven applications aimed at automating the extraction of diagnostic information from imaging data.

The development of radiomics studies requires a deep understanding of the complex machine learning (ML) pipeline, which often goes beyond the expertise of a single radiologist. However, considering that the integration of AI in radiology is expected to have significant professional implications [6,7,8], it is essential for radiologists to acquire foundational skills in this field, which will allow them to critically evaluate the impact of these technologies in daily clinical practice and to strengthen their collaborative role with data scientists [9].

While many comprehensive reviews provide an introductory overview of radiomics aims and applications [10,11,12,13], very few have ever addressed the mechanisms and challenges of the ML workflow in a unified framework [8,14,15,16,17,18,19].

Therefore, this paper pursues two key objectives. First, it aims to offer an introductory but rigorous guide to the core ML concepts applied to radiomics. Second, it aims to provide a roadmap for navigating the recently proposed Methodological Radiomics Score (METRICS, 2024) tool [20] for evaluating the quality of radiomics studies, helping researchers and reviewers adopt it for study assessment. METRICS offers expert opinion-based importance weights for categories and items, marking the first time a scoring tool has adopted such a transparent methodology. The tool is versatile, covering both hand-crafted radiomics and fully deep learning-based pipelines, adapting to various use cases [20].

The structure of this article is as follows: Section 2 presents a brief overview of ML and deep learning (DL) definitions; Section 3 introduces the bias–variance trade-off, the cornerstone of any model optimization process; Section 4 dissects the various stages of the radiomics study pipeline, including study design, image preprocessing, feature selection, model training, validation, and performance assessment; and Section 5 discusses some of the latest developments and highlights potential challenges in the evolution and implementation of these tools.

**Figure 1:**
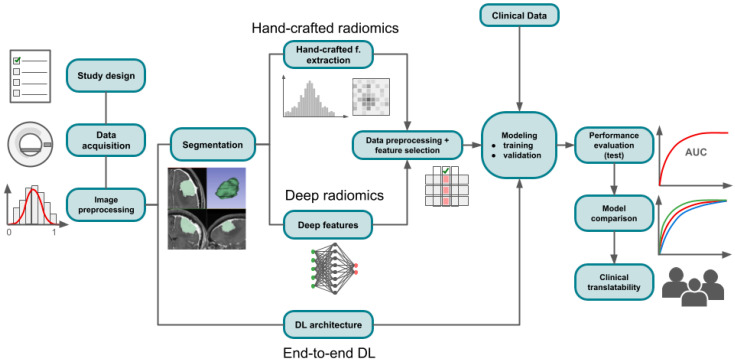
Overview of the radiomics framework.

In general, a radiomic approach can follow one of three different pathways: (1) hand-crafted radiomics, (2) deep radiomics, or (3) end-to-end deep learning (Figure 1).

**Figure 2:**
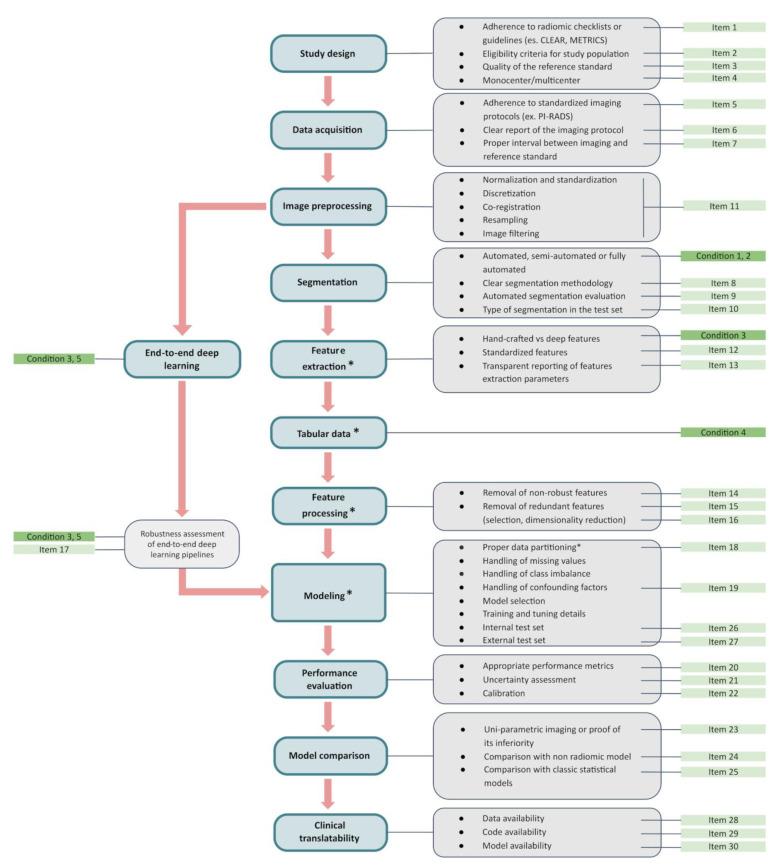
The main steps, the specific sub-tasks for each phase, and the related METRICS items. * To avoid data leakage and overfitting, data partitioning should be performed before feature extraction, processing, and model training.

The METRICS tool incorporates conditional items designed to cover all of these pathways [13,14,15]. A detailed, step-by-step roadmap is presented in Figure 2, including subtasks specific to each phase and the corresponding items of the METRICS tool. Table 1 lists METRICS items and the corresponding paragraphs in which the topic is discussed.

In hand-crafted radiomics, mathematically designed imaging features are extracted from the segmented region or volume of interest and presented as a tabular dataset for modeling using traditional statistical ML models or neural networks. In contrast, deep radiomics employs DL models, particularly convolutional neural networks, to automatically learn and extract features from images. The end-to-end DL approach integrates the entire image processing pipeline into a DL model, directly predicting outcomes from raw images without manual intervention.

## 2. Machine Learning and Deep Learning

Radiomics extensively utilizes ML models [4,8,21]. These models leverage computational power to automate algorithms that learn from data, mimicking human learning processes and improving accuracy over time through experience [22,23,24,25]. More specifically, ML algorithms encompass a wide range of approaches for estimating functions that map the relationship between a set of inputs, X, and an output variable, Y. According to the context, input variables are also referred to as predictors or independent variables, while output variables can be called the dependent variable, target, label, or response [25]. In radiomics, the predictors always include imaging features and may also incorporate clinical, demographic, or laboratory variables. Conversely, the target variable can be either quantitative (e.g., months of survival) or qualitative (e.g., histological subtype or recurrence prediction).

**Figure 3:**
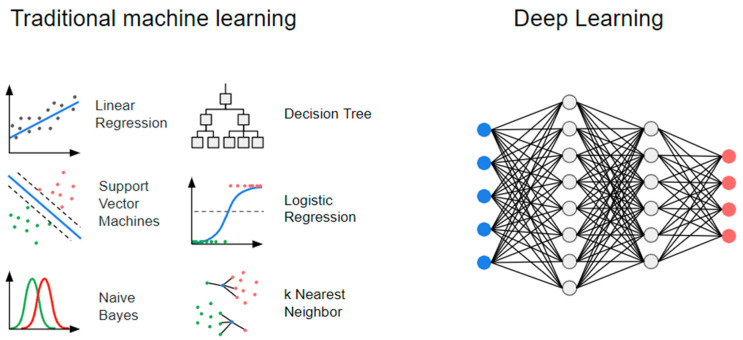
Machine learning and deep learning.

ML algorithms can be divided into traditional (“statistics-based”) ML models and neural networks (Figure 3).

Traditional ML models can be seen as the automation of classical statistical approaches and their derivatives, which are typically represented by mathematical formulas [25], such as the regression line of linear regression: (f(X) = β0 + β1X1 + β2X2 + … + βpXp) [20]. Other examples include logistic regression, decision tree (DT), support vector machines (SVM), naive Bayes, and k-nearest neighbor (KNN). In general, traditional ML models require less training data and offer greater interpretability and transparency, providing clearer insights into how input variables influence the output [26]. However, their capacity to capture complicated patterns is limited [25,26,27,28].

On the other hand, neural networks follow a completely different paradigm, inspired by the biological structure of the human brain [27,28]. These models consist of layers of nodes (neurons) that process data through weights and activation functions [27,29]. When neural networks contain a large number of intermediate layers, they are referred to as deep neural networks [27,29]. Deep learning (DL) employs these deep neural networks, allowing for more complex non-linear representations at the cost of a more difficult interpretation of their internal organization, i.e., the “black box” phenomenon [27,28,29].

Convolutional neural networks (CNNs), a specific type of deep neural networks designed for image analysis, use convolutional layers that compare overlapping regions of the input with small weight matrices, known as kernels or filters, to encode image features. While increasing the number of layers enhances their ability to learn complex patterns, it also makes these DL models harder to train [27,28]. This has led to architectural refinements, such as skip connections and bottleneck blocks (as seen in ResNet), multibranch convolutions (GoogLeNet), and ensemble methods [29]. CNNs have been widely adopted in radiomic studies, and they can be used in an end-to-end fashion or to support specific subtasks of the radiomics pipeline, such as data augmentation, feature extraction, image segmentation, classification, prediction, or multimodal integration.

## 3. Bias–Variance Trade-Off

The trade-off between bias and variance is key in ML, as many of the challenges and methodological issues in ML ultimately aim to optimize this balance [25,30]. Bias measures the average difference between the predicted values and the true values, reflecting how accurately a model can predict on the training dataset. High bias indicates that the model is not capturing the underlying patterns of the data. On the other hand, variance measures the variability of model predictions across different datasets, indicating how well the model can generalize to new, unseen data. High variance means the model is overly sensitive to the training data, resulting in more errors on the testing set (i.e., new, unseen data). Essentially, bias and variance inversely represent model accuracy for training and test sets, respectively.

**Figure 4:**
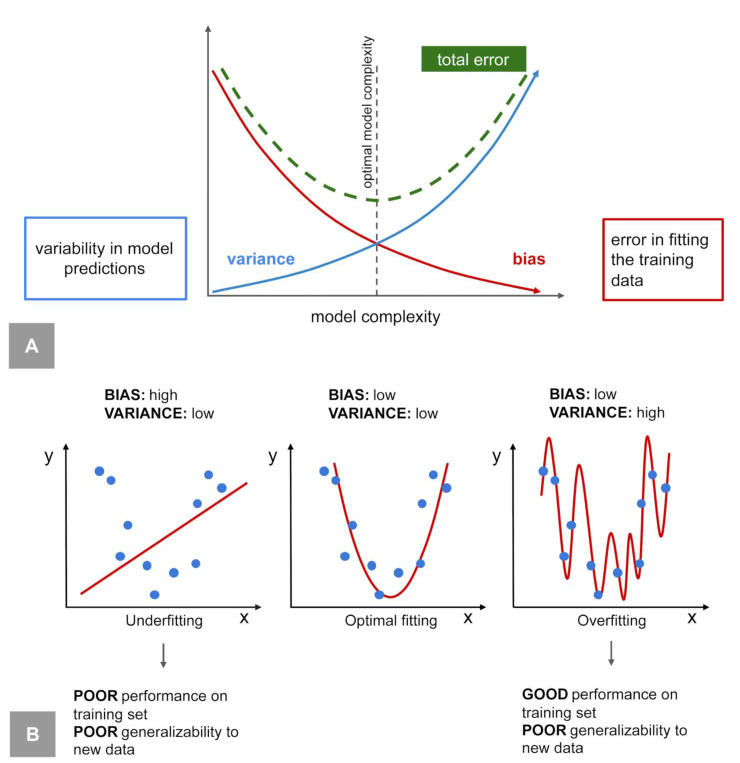
Bias–variance trade-off, overfitting, and underfitting. (**A**) Bias–variance trade-off. As the complexity of the model increases, the bias decreases but the variance increases. More complex models can capture intricate patterns in the data, better fitting the training dataset and reducing systematic errors (bias), but they also become more sensitive to noise and specific data points, leading to higher variability in model predictions (variance). (**B**) Overfitting and underfitting. Overfitting occurs when a model captures noise and fluctuations in the training data rather than the underlying patterns, resulting in an excellent performance on the training set (low bias) but poor generalizability to new data (high variance). Underfitting happens when a model is too simple to detect the underlying patterns, leading to a poor performance on both the training (high bias) and testing datasets.

As shown in Figure 4A, as the complexity of the model increases, the bias decreases while the variance increases. Although developers aim to reduce both, it is often not possible to do so simultaneously, leading to the necessity for regularization, which decreases the model variance at the cost of increased bias.

The concepts of bias and variance are strictly related to overfitting and underfitting (Figure 4B) [14,25]. Overfitting occurs when the model has low bias but high variance, capturing noise and fluctuations in the training data rather than the underlying pattern. By introducing regularization and feature selection, the model’s variance ***reduces*** and the model’s performance on unseen data improves, at the cost of slightly increasing the model’s bias. Underfitting occurs when a model has high bias and ***low*** variance, resulting in a poor performance for both training and testing data. This often happens when the model is too simple or the training data are insufficient.

**Figure 5:**
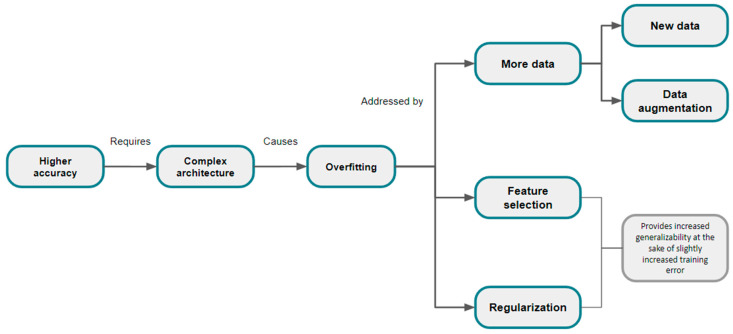
Strategies for addressing the problem of model overfitting.

Constructing models with good performance necessitates a large number of predictors [15,25,30,31,32]. However, this complexity can lead to overfitting, creating a disadvantageous loop where the model fits the training data too closely and performs poorly on unseen data. Overfitting can be mitigated by several methods (Figure 5), discussed in the following paragraphs.

## 4. Step-by-Step Radiomic Workflow

### 4.1. Study Design and Data Collection

When formulating a radiomics study, adherence to established guidelines and checklists—such as CLAIM [33,34] and CLEAR [27]—is crucial for ensuring rigorous design and reporting standards (METRICS item 1 [20]).

#### 4.1.1. Eligibility

Eligibility criteria must be meticulously defined to select a representative sample of the population of interest. Therefore, inclusion and exclusion criteria should be explicitly outlined to minimize bias (METRICS item 2 [20]).

#### 4.1.2. Reference Standard

A high-quality reference standard is essential for robust outcome measures (METRICS item 3 [20]) [28]. This standard should align with current clinical practices and reliable methods, such as histopathology, well-established clinical scores, genomic markers, prognostic tools, or consensus-based guidelines and expert opinions [20].

Furthermore, the time interval between the examination and the acquisition of the reference standards must accurately reflect the presence or absence of target conditions at the time of the diagnostic exams (METRICS item 7 [20]) [11,15]. If there is a significant delay, changes in the patient’s condition or disease progression could undermine the validity of the radiomic analysis.

#### 4.1.3. Monocentric Versus Multicenter

Radiomic analysis relies heavily on the quality and consistency of diagnostic imaging data [14,16,35]. Indeed, the involvement of various institutions (METRICS item 4 [20]) is beneficial to enhance the generalizability and robustness of the radiomic models. However, a multicenter design does not necessarily imply external validation. These are two separate design choices, even if one (availability of different data sources) is a prerequisite of the other (external validation/testing). Of note, a multicenter setting implies that the variability of imaging protocols between different institutions must be carefully managed to prevent distortions [14,15,36,37].

#### 4.1.4. Imaging Protocol

The adherence to established guidelines for imaging acquisition protocol is essential for ensuring consistency and comparability across different imaging studies, as well as clinical translatability (METRICS item 5 [20]) [11]. For example, in clinical practice for prostate cancer, the PI-RADS guidelines provide specific recommendations for MRI acquisition parameters, ensuring that obtained images are of a high quality and suitable for accurate assessment of prostate lesions [38,39]. Therefore, acquisition protocols should be clearly reported (METRICS item 6 [20]), as standardized acquisition protocols reduce the variability in image quality and technical parameters, which can significantly impact the extracted radiomic features. Whenever possible, preference should be given to single imaging sets (such as a single MRI sequence) over multi-parametric imaging to avoid unnecessary data complexity and overfitting [20].

### 4.2. Image Preprocessing

Preprocessing medical images is a crucial step that impacts the accuracy and reliability of feature extraction (METRICS item 11 [20]). Preprocessing is essential for reducing variability and making radiomic features comparable across different scans and patients [40,41,42], especially considering the diverse imaging modalities (e.g., PET, MRI, and CT) and the specific requirements of different feature extraction techniques (e.g., 2D versus 3D). Image preprocessing encompasses several procedures that are outlined in the following subsections [43].

#### 4.2.1. Normalization and Standardization

In image preprocessing, normalization and standardization are often confused but are distinct processes. Normalization (or min–max scaling) scales data to a specific range, like [0, 1] or [−1, 1], while standardization (or Z-score normalization) adjusts data to have a mean of 0 and a standard deviation of 1, transforming them to a normal distribution [44], allowing a better understanding of the data’s spread and variability [14,16]. Both are used to prepare data for algorithms, facilitating optimal comparisons across data acquisition methods and texture instances [45].

Normalization entails dividing each pixel’s value by the maximum possible value for that pixel (255 for an 8 bit image, 4095 for a 12 bit image, and 65,535 for a 16 bit image). For instance, CT images are primarily encoded in 12 bits with gray levels; normalizing a CT image involves dividing each pixel value by 4095 to achieve a range between 0 and 1 [46].

Standardization is especially recommended in MRI since, unlike a CT, units of signal intensity are arbitrary [16].

Different normalization methods impact feature extraction, and the choice of method depends on dataset characteristics and desired feature types [47].

#### 4.2.2. Discretization

Discretization involves modifying images to adhere to a common format or scale and is crucial for MRI scans, where intensity values can differ significantly across different scanners or scanning protocols [48,49,50,51,52]. Gray-level discretization consists of converting continuous intensity values (or density for CT) into discrete bins, simplifying the intensity distribution and facilitating texture analysis. Discretization involves three key parameters: the range of the data, the number of bins, and the width of each bin, but only two can be independently controlled. The range is typically preserved from the original data, although it may be adjusted in certain contexts, such as when comparing it to a reference dataset. The optimal bin number in discretization is crucial, as too few bins can obscure features, while too many can amplify noise, and finding the right balance depends on both data acquisition parameters and feature content [15]. Fixing the bin number, as often done in MRI, helps normalize image intensities and improve reproducibility across different samples. In contrast, fixing the bin size, as often done in PET, allows for a direct relationship between the bins and the original intensity scale, aiding in the comparison of data with different ranges [15]. The parameters for discretization, such as bin width and the resulting gray-level range, or bin count, should be thoroughly reported [48].

#### 4.2.3. Co-Registration

Image co-registration is necessary when a single mask is to be applied to all image sets (e.g., multiple contrast phases or sequences), ensuring that the anatomical structures are correctly aligned [53]. This process involves aligning images from different time points, scanners, or imaging modalities to a common coordinate system. In neuroimaging studies, the co-registration of images may also involve aligning images from different sequences or modalities to a common reference frame using anatomical atlases, like the one from the Montreal Neurological Institute, used to map the location of brain structures, regardless of individual differences in the overall brain size and shape [54,55].

#### 4.2.4. Resampling

Upsampling and downsampling are techniques used to adjust the image resolution [15]. In cases of a large slice thickness (e.g., ≥5 mm), extreme upsampling to a very fine resolution (e.g., 1 × 1 × 1 mm^3^) might introduce artifacts and inaccuracies [56]. Instead, using 2D feature extraction techniques that ensure in-plane isotropy of the pixels can be more appropriate [20,56]. Conversely, for 3D feature extraction, achieving isotropic voxel values is essential for ensuring the rotational invariance of texture features [57].

#### 4.2.5. Image Filtering and Enhancement Techniques

Image filtering and enhancement techniques are used to highlight specific features or to reduce noise [14,16,58]. Image filtration can be used before the extraction of features as a preprocessing step to highlight particular image properties. For instance, wavelet decomposition uses low-pass filters to capture low-frequency components (basic structures of the image) and a high-pass filter to capture high-frequency components (fine details, such as edges) [59]. Moreover, Laplacian of Gaussian filtering, with specified σ values, can enhance edges and make the features more distinct [60]. It is important to document the type of filters used and their parameters, as these can significantly affect the extracted features.

### 4.3. Segmentation

In computer vision, image segmentation involves clustering together parts of an image that belong to the same object class, thus creating a region of interest (ROI) in 2D images or a volume of interest (VOI) in 3D images [14,15,16,61,62]. Indeed, this process enhances the relevance of the segmented areas, making them easier to interpret and analyze.

The METRICS tool encompasses a wide definition of segmentation, including (1) fine delineation of a ROI or a VOI; (2) rough delineation with bounding boxes; or, (3) cropping the image around a ROI (METRICS condition 1 [20]), the last two mostly applying to deep radiomics. In radiomics studies, the ROI/VOI generally coincides with a mass or nodule (for example, a lung tumor), but it can also be a tissue or an organ [62,63].

There are several segmentation methods, each with its advantages and limitations [64]. Based on the level of human intervention, they can be classified as manual, semi-automatic, and automatic (METRICS condition 2 [20]). According to the METRICS tool, any manual adjustments to the annotation, in terms of area, volume, or predefined parameters, classify the technique as semi-automatic [20]. The segmentation procedure should be explicitly detailed (METRICS item 8 [20]), as accurate tumor segmentation is one of the primary challenges for ensuring the reliability of radiomic features, particularly when using manual or semiautomatic methods.

**Figure 6:**
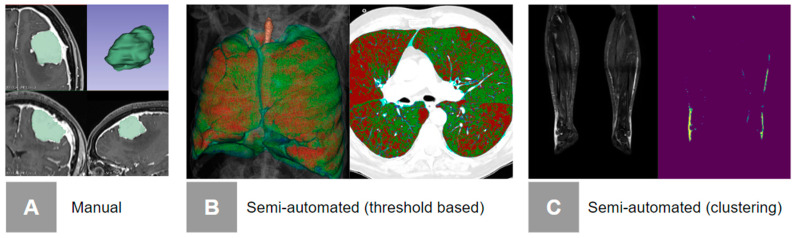
Examples of automated and semi-automated segmentation methods. (**A**) Fully manual segmentation. (**B**) Threshold segmentation. For example, a threshold of −950 HU is set to automatically segment (in red) the areas of emphysema [65]. The human annotator checks for the accuracy of the segmentation process and might adjust for inaccuracies; in this case, the process is called semi-automatic. (**C**) Clustering-based segmentation. Unsupervised clustering algorithm (k-means) is used to segment the signal associated with lymphedema and to differentiate the lymphedema from the signal of other types of tissue.

Fully manual segmentation, where the human annotator manually outlines the ROI (Figure 6A), represents the simplest but most time-consuming option, and is typically performed when dealing with small datasets that can be annotated by expert radiologists [14]. Its other main limitation lies in the frequent absence of standardized segmentation protocols, with high intra- and inter-observer variabilities [14,16].

Currently, several open source and proprietary software solutions allow for the automatic or semi-automatic segmentation of radiological images. Among the most popular are 3D Slicer, MITK, ITK-SNAP, LifEx, and ImageJ [15,16,66].

Semi-automatic segmentation typically involves utilizing customized algorithms to segment images based on predefined parameters, after which manual verification and adjustments are made by an expert to maximize accuracy [67]. Semi-automated segmentation techniques include thresholding, edge detection, and region-based segmentation [64]. Thresholding techniques classify pixels based on their intensity values; they are simple and fast, but may struggle with images that have poor contrast (Figure 6B) [64,65,68]. Edge detection methods identify boundaries within an image, providing a clear delineation of structures, though they can be more sensitive to noise [69]. Region-based segmentation groups neighboring pixels with similar properties, resulting in more homogeneous regions, but potentially missing fine details. Clustering algorithms, such as k-means or hierarchical clustering, categorize pixels into distinct clusters based on their attributes (Figure 6C) [70].

Deep neural networks are extremely powerful tools used to perform automatic segmentation and can reduce the workload and increase reproducibility in the setting of hand-crafted radiomics [71,72]. For example, encoder–decoder architecture, pioneered by the fully convolutional network, is commonly used for segmentation tasks [27]: the encoder downsamples the image through convolutional layers, while the decoder upsamples the feature maps to produce per-pixel labeled outputs. U-Net, a popular architecture for semantic segmentation, employs a symmetric U-shaped design with skip connections to facilitate upsampling. For the so-called “instance segmentation”, which combines object detection and semantic segmentation, the Mask R-CNN architecture is prominent: it extends two-stage detection models by adding a branch to predict binary masks for each object category, enabling instance segmentation [71]. The primary limitations of DL-based automatic methods stem from their requirement for large, labeled datasets to train accurate models, as well as concerns about the generalizability of these algorithms, which may perform poorly when applied to datasets different from those used during training [14].

If an entirely automated segmentation technique is used, examples of the results should be provided, and a formal accuracy assessment should be included in the study, comparing the results with those of expert annotators (for example, using the DICE score or Jaccard index against a radiologist’s semantic annotation) [73,74]. This requirement also applies to the use of segmentation models that have been previously validated on other datasets (METRICS item 9 [20]).

In any case, a clear statement should be provided about whether the final segmentation in the test set is produced by a single reader (manually or with a semi-automated tool) or an entirely automated tool (METRICS item 10 [20]).

### 4.4. Feature Extraction

Radiomic features are quantitative characteristics extracted from medical images and represent the main input of the radiomic model [4,5,16,75]. These features represent the radiological “signature” of phenotypes or biological characteristics and can be used in ML modeling for diagnostic, prognostic, and predictive purposes [76,77,78,79]. Radiomic features can be categorized into two main types: handcrafted features and deep features [14,15,20,78].

#### 4.4.1. Hand-Crafted Features

Radiomic features are termed “hand-crafted” because they are generated by algorithms designed or selected by data scientists, rather than being learned directly from images as in deep learning (METRICS condition 3 [16,20]).

Hand-crafted features are traditionally divided into first-order, second-order, and higher-order features (Table 2) [80,81,82]. As the order increases, they become less interpretable, meaning it becomes more difficult to visually understand what they represent in the images. First-order features describe the distribution of values of individual voxels disregarding their spatial relationship (for example, mean, median standard deviation, kurtosis, etc.), or basic geometric properties of the ROI/VOI, such as volume and maximum surface area [81,83]. Second-order features, for example those derived from the gray-level co-occurrence matrix, capture textural information by examining the spatial relationship between pixel pairs [84]. Higher-order features provide more complex textural patterns and spatial relationships within the image [80]. For a list of hand-crafted features, see [19]. There is no general consensus on the definition, giving rise to problems in comparing different radiomics studies [19]. Some authors categorize radiomic features into different classes based on their significance [81]. These include intensity-based measures (primarily first-order features), heterogeneity and texture (second-order features), shape and volume (second-order features such as volume, sphericity, compactness, and surface-to-volume ratio), peritumoral radiomics (which assesses structural heterogeneity in the peritumoral region surrounding a tumor, including stroma, lymph nodes, and potential metastatic sites), and tumor vascularity radiomics (vessel tortuosity and structural organization).

The feature classes and the number of extracted features for each class should be clearly reported (METRICS item 13 [20,34]).

The Image Biomarker Standardisation Initiative (IBSI) provides guidelines for the extraction, definition, and validation of hand-crafted radiomic features [85]. This standardization ensures reproducibility and comparability across different studies and institutions, thereby enhancing the reliability and clinical utility of radiomic analyses. Whether the feature extraction was conducted according to a compliant standard is the subject of the METRICS item 12 [20].

#### 4.4.2. Deep Features

On the other hand, deep features are automatically extracted using DL methods, capturing patterns and abstractions without explicit human intervention [21,27]. Since DL methods utilize CNNs, their deterministic output helps eliminate variations both within and between observers [14,21]. Compared to hand-crafted features, deep feature representation works at the expense of less interpretability of the geometric and radiological meaning of individual features [27,86]. In the case of DL, the architecture of the neural network should be described along with all operations on the image (METRICS item 13 [20]).

Deep features can be “exposed” to the user and available for processing as tabular data.

### 4.5. Tabular Data

After the extraction of hand-crafted features from medical images, the results are organized into a tabular data structure, often referred to as a “tabular dataset”. This step applies to both hand-crafted radiomics and deep radiomics, but not to the end-to-end deep learning pathway (METRICS condition 4 [20]). This tabular dataset consists of rows and columns, where each row represents an observation (ROI) and each column represents a radiomic feature or other clinical and demographic variables used as predictors [15]. A tabular structure improves data manipulation and analysis, allowing researchers to perform better data cleaning and statistical computations [87].

### 4.6. Data Preparation: Missing Values, Data Scarcity, Confounding Factors, and Class Imbalance Problems

The accuracy of radiomic models can be undermined by data scarcity, confounding factors and class imbalance problems, which can introduce biases and lead to unreliable predictions [88,89,90]. Handling confounding factors and class imbalance issues can span across both data preparation and modeling; however, the primary efforts to address these issues occur during data preparation.

#### 4.6.1. Missing Values

Handling missing values is crucial for maintaining the integrity and validity of analyses [34]. Missing values can arise from several sources, such as image acquisition errors, technical problems, or simply from variability in clinical practices [91]. Strategies to handle missing data include removing records with missing values, simple imputation methods in which missing values are replaced by statistics, like the mean or median, and advanced imputation techniques exploiting ML approaches [90,92]. Additionally, analyzing missing data patterns to determine if they are random or systematic can prevent bias and ensure robustness and reliability [93].

#### 4.6.2. Data Scarcity

Radiomic datasets can be subject to limited sizes and a scarcity of data that easily results in overfitting. This problem can be addressed through several approaches. Data augmentation consists of applying a series of transformations to the original images to obtain new data [93]. Generative adversarial networks (GANs) are a peculiar type of DL network particularly suitable for this purpose, capable of creating “fake” images from the originals [26]. Conditional GANs introduce additional input information to guide the generation process, while CycleGANs translate images from one domain to another, useful for tasks such as medical image synthesis and translation [94,95]. These approaches can also be used to evaluate the robustness of radiomics features in different conditions.

#### 4.6.3. Confounding Factors

Confounding factors are variables that independently affect both the predictors (radiomics features) and the target variables (disease outcomes), misleading the model into attributing effects to the radiomic features that are actually due to the confounders [89,96]. For instance, in a study aiming to predict disease outcomes based on certain imaging features, age and socioeconomic status might act as confounders if not appropriately controlled. Since the variability of imaging exams is typically controlled in early stages (imaging protocol and preprocessing), confounding factors primarily rely on different distributions of demographic or clinical features (e.g., sex, stage, or lesion grade) across sites or scanners. Their presence can lead to spurious associations and erroneous conclusions; therefore, the proper handling of confounding factors is the key to developing a robust radiomic pipeline (METRICS item 19 [20]).

To mitigate the impact of confounding factors in clinical studies, several strategies can be employed, like stratification, matching, statistical adjustment, and randomization [89]. However, in retrospective contexts, as is generally the case of radiomic studies, researchers must rely on confounding control techniques during data analysis, rather than during collection. These include statistical methods such as multivariate analysis, propensity score matching, and others [97,98]. However, these techniques can only partially mitigate the impact of confounders, and their success depends on the availability and quality of information about potential confounders.

#### 4.6.4. Class Imbalance Problems

Class imbalance occurs when the number of instances in one class significantly outnumbers the instances in other classes [99,100]. This imbalance can skew the performance of ML models, leading to biased predictions that favor the majority class. For example, if a dataset contains 95% healthy cases and only 5% diseased cases, a model that predicts every case to be healthy would achieve high overall accuracy, despite being practically useless. To address class imbalance problems oversampling, undersampling, cost-sensitive learning, algorithmic approaches, and ensemble methods [100].

Addressing both confounding factors and class imbalance often requires an integrated approach. For instance, while stratifying the data to control for confounders, one might also need to apply resampling techniques within each stratum to address class imbalance. Additionally, preprocessing steps should be carefully designed to ensure that solutions to one problem do not exacerbate the other.

### 4.7. Features Robustness

Feature robustness refers to the stability (or reliability) of radiomic features when subjected to variations in imaging conditions, such as different scanners, acquisition parameters, and image preprocessing techniques [101,102,103]. When evaluating the robustness of radiomics features, the aim is to determine how much of the feature variability is attributable to the intrinsic properties of the imaged object (such as a tissue or a tumor) and how much is due to the methods of image acquisition.

**Figure 7:**
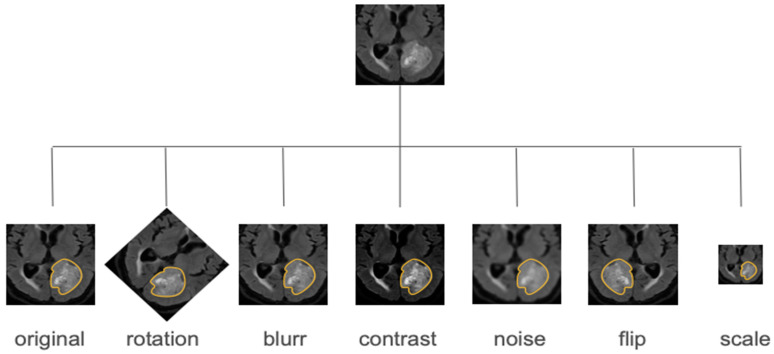
Examples of data augmentation techniques.

Methods for testing robustness include test–retest analysis, where images of the same subject are acquired at different times, and image perturbation, where the original image is subjected to a series of transformations, such as adding noise, translation, rotation, and others (Figure 7) [94,104].

Factors that can affect robustness include noise, resolution, segmentation variability, ROI size, but also image preprocessing steps, such as normalization and resampling [50,105]. Ensuring robustness is crucial for the clinical applicability of radiomic features (METRICS item 14 [20]) [20,106,107].

The Intraclass Correlation Coefficient (ICC) and the Concordance Correlation Coefficient (CCC) are two common metrics used for assessing the robustness of radiomic features (Table 3) [20,108,109]. The ICC and CCC offer a comprehensive evaluation of feature robustness, assisting researchers with identifying features that are less influenced by technical and biological variations. Features with a high CCC (i.e., >0.9) and ICC (i.e., >0.75) demonstrate strong test–retest reliability and inter-observer agreement, respectively, ensuring high reproducibility [14,20,21,110]. These features are subsequently retained for further analysis, while features falling below these thresholds are discarded [14,20,21,110].

Assessing the consistency of performances in an end-to-end DL pipeline is essential for ensuring its robustness and clinical reliability. This can be achieved in practice by taking advantage of some of the approaches discussed above, including test–retest settings, such as scan–rescan studies, segmentations by different readers, or stability analysis involving image perturbations. These evaluations help determine whether the model maintains high performance, despite variations in the imaging conditions or reader interpretations (METRICS item 17 [20]).

### 4.8. Feature Selection and Regularization

#### 4.8.1. The Need for Feature Selection and Regularization

Not all extracted features are useful for modeling, hence the need for feature selection (METRICS item 15 [20]) [111], which must be performed solely on the training set. A critical problem in radiomics studies is the high dimensionality of datasets which leads to: (1) a large computational demand and (2) an excessive number of features that can cause a reduction in model performance due to overfitting. Therefore, feature selection methods are used to simplify the model by removing redundant and irrelevant ones [84]. The subset of features obtained through feature selection should be minimal but effective in accurately identifying the target and, ideally, should improve the accuracy of the prediction model [84]. In the process of mapping high-dimensional data into a low-dimensional space, however, there is a risk of losing some important information; for this reason, the choice of the method is a crucial step in the pipeline of a radiomics study [112]. While in traditional radiomics feature selection is an explicit and crucial phase, in deep radiomics it is often incorporated into the learning process of the model itself.

#### 4.8.2. Filter, Wrapper, and Embedded Methods

**Figure 8:**
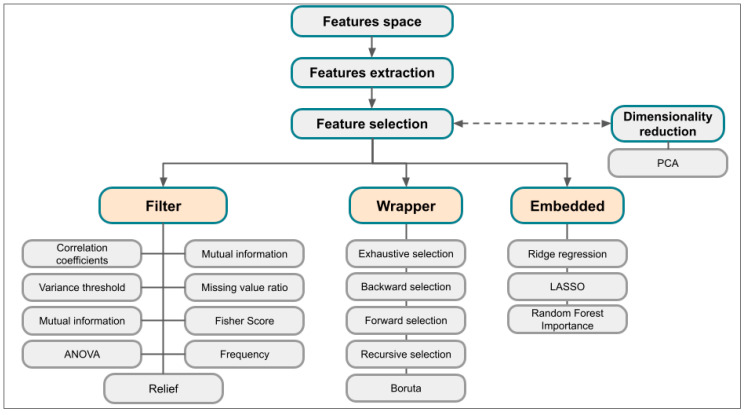
Overview of feature selection methods: filter, wrapper, and embedded.

**Figure 9:**
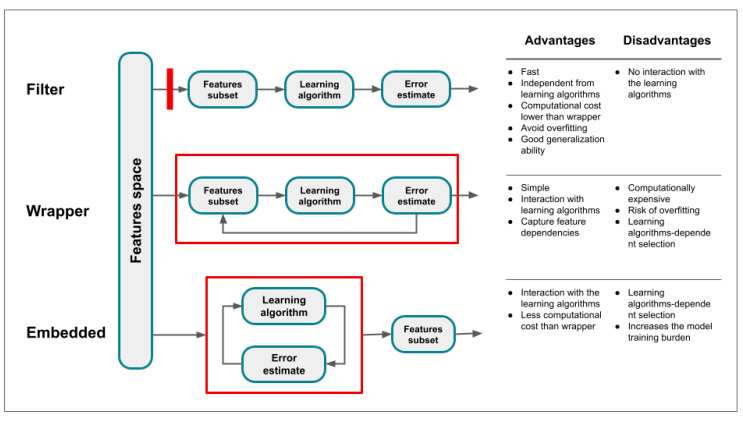
Mechanism of feature selection methods, advantages, and disadvantages.

Feature selection methods can be distinguished based on the relationship with the learning model in filter, wrapper, and embedded methods (Figure 8 and Figure 9) [100].

**Figure 10:**
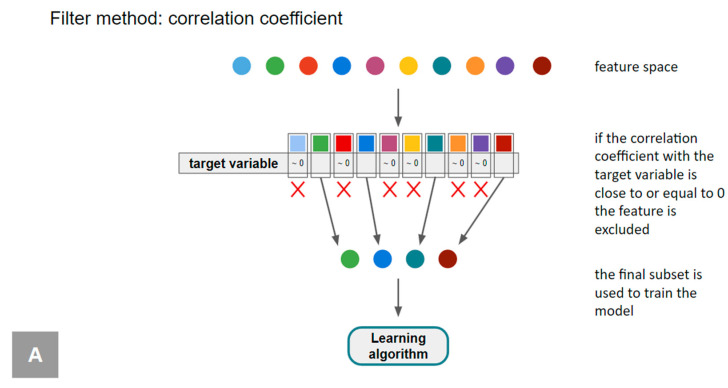

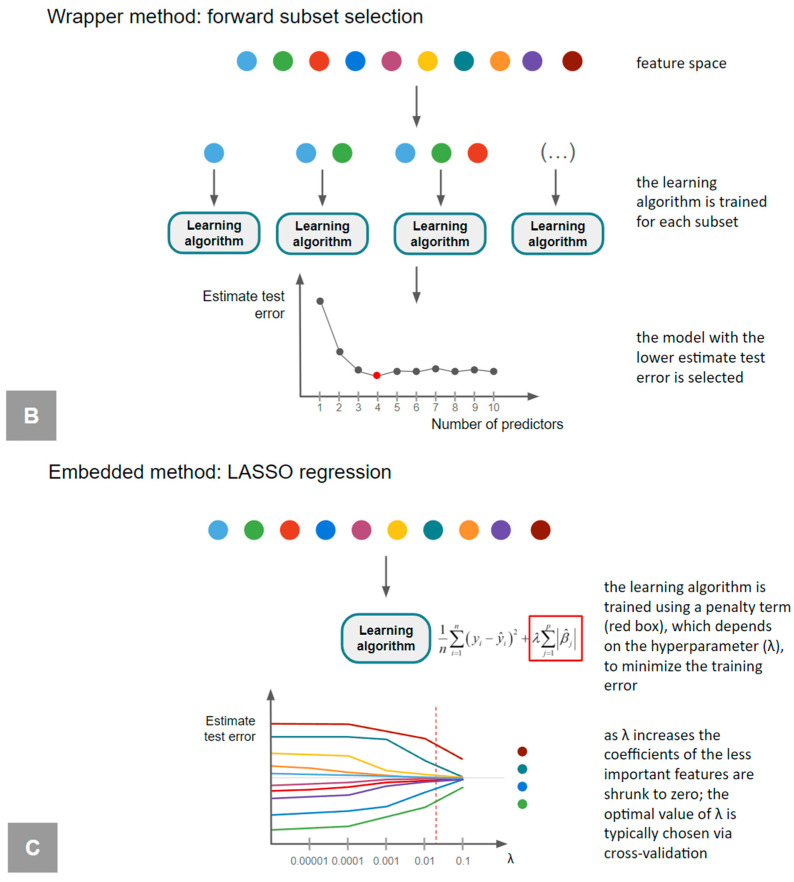
Examples of feature selection methods. (**A**) Filter method: correlation coefficient. (**B**) Wrapper method: forward subset selection. It starts with 1 predictor and gradually adds one predictor at a time until the optimal model is achieved. The estimated test error can be calculated indirectly by adjusting the training error to account for the bias due to overfitting, or directly for example through cross-validation. In this example, the minimum estimate is achieved using 4 predictors. (**C**) Embedded method: least absolute shrinkage and selection operator (LASSO) regression. It is a type of linear regression that incorporates regularization to enhance the model’s prediction accuracy and interpretability. It achieves this by adding a penalty term to the ordinary least squares (OLS) objective function, which is the sum of the absolute values of the model coefficients. This penalty term encourages the coefficients of less important features to shrink toward zero. As a result, lasso regression not only performs regularization but also variable selection, making the model more interpretable by reducing the number of features. Lasso regression is particularly useful when dealing with high-dimensional data, where the number of predictors can be large. It helps prevent overfitting by reducing model complexity and can improve the model’s prediction accuracy for new, unseen data. However, choosing the appropriate value for λ is crucial, typically achieved through cross-validation.

In filter methods, features are evaluated and ranked based on statistical criteria using predefined thresholds for the chosen statistical measure, independently of any learning algorithm [112,113,114,115] (Figure 9 and Figure 10A, Table 4). This evaluation is performed directly from the tabular data using measures such as correlation coefficients, mutual information, and the χ^2^ test [25,113,114,115]. When the correlation coefficients are close to or equal to 0 with the target variable, the feature is excluded, as this indicates that it does not significantly contribute to explaining the variations in the target variable. The main advantage of this method is that it is not biased toward any specific learner model and maintains a simple structure.

Wrapper methods use feedback from the learning algorithms to guide feature selection (Figure 10B) [84]. First, specific rules are applied to generate candidate feature subsets. Then, the optimal subset is selected by comparing the performance of models trained on different subsets. Wrapper methods include best subset selection, stepwise model selection, and others (Table 5) [116,117]. The best subset of features can be achieved through an exhaustive search that consists of testing all *2p* possible models containing subsets of the *p* predictors. The main disadvantage of this method is that the increase of combinations leads to an exponential increase in computational costs [25]. Forward and backward stepwise selections are both computationally efficient alternatives (Figure 10B).

Embedded methods combine the advantages of filter and wrapper methods by integrating feature selection directly into the model construction process. This approach eliminates the need for multiple runs of the learning model [113]. Shrinkage methods are a type of embedded method that fit a model using all predictors, but the estimated coefficients are progressively shrunken toward zero depending on a penalty parameter. This process, also known as regularization, reduces variance at the cost of slightly increasing the bias, with the result of significantly improving the model’s performance for new unseen data [76]. According to the type of shrinkage performed, some coefficients may be estimated as exactly zero, allowing these methods to also perform variable selection. In this case, after the feature selection process is finished, the model is trained. A common embedded method is the least absolute shrinkage and selection operator (LASSO) [118], a type of linear regression that incorporates regularization to enhance the model’s prediction accuracy and interpretability (Figure 10C). It achieves this by adding a penalty term to the ordinary least squares objective function, which is the sum of the absolute values of the model coefficients. This penalty term encourages the coefficients of less important features to shrink toward zero. As a result, LASSO regression not only performs regularization, but also variable selection, making the model more interpretable by reducing the number of features. LASSO regression is particularly useful when dealing with high-dimensional data, where the number of predictors can be large. It helps prevent overfitting by reducing model complexity and can improve the model’s prediction accuracy for new, unseen data. However, choosing the appropriate value for λ is crucial and is typically achieved through cross-validation.

#### 4.8.3. Dimensionality Reduction

The optimal number of the subset of features is controversial and depends on the context; generally, the “rule of thumb” of a maximum of ten features for each instance is considered (METRICS item 16 [16,20,34]).

Dimensionality reduction techniques should be mentioned, the best known being principal component analysis (PCA) [20]. PCA maps the original feature space to new coordinates, called principal components, which are linear combinations of the original features, generating a new data representation that preserves the maximum possible variance. Therefore, PCA does not select specific features from the original ones; rather, it transforms the entire set of features into a set of principal components representing the directions of maximum variance in the data [112].

### 4.9. Modeling

#### 4.9.1. Supervised Versus Unsupervised Learning

**Figure 11:**
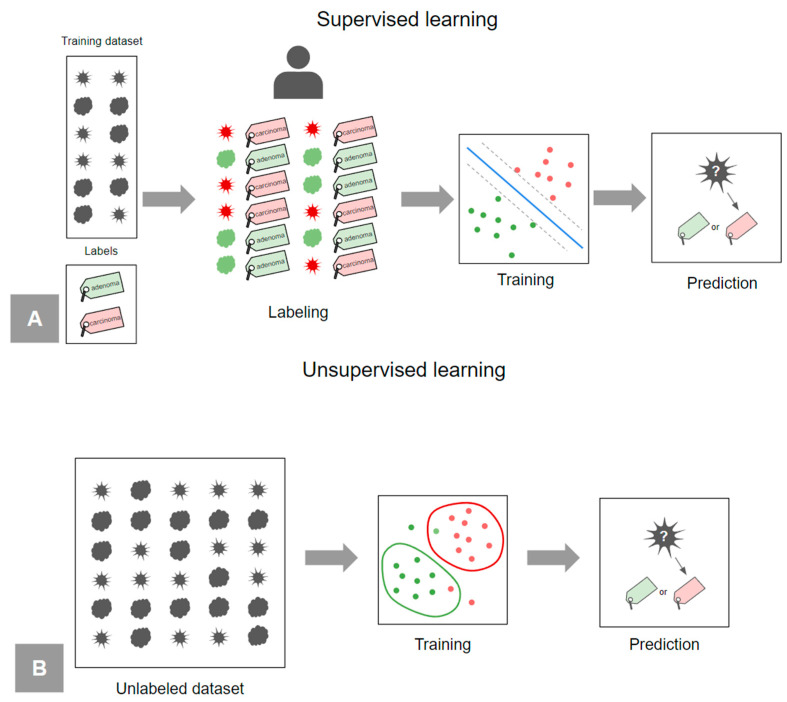
(**A**) Supervised learning. (**B**) Unsupervised learning.

Supervised learning is a type of ML in which the model is trained on a labeled dataset, which means that each observation is associated with a known value of the output [25,30]. For example, each subset of features representing a lesion comes with an associated label (malignant/benign) (Figure 11A). The supervised model learns to map from inputs to outputs in order to correctly predict the labels for new unseen data. In practice, the label of the target variable can be assigned by the radiologist, if the imaging diagnosis is adopted as the reference standard, or by the pathologist, if the histopathological diagnosis is adopted. Most radiomics studies fall into this paradigm, and many ML models work in a supervised framework.

On the other hand, unsupervised learning describes a more challenging situation in which the output associated to each observation is unknown [25,30]. In this scenario, for example, it is not possible to fit a linear regression model, since there is no output variable to predict. Unsupervised algorithms extract knowledge directly from the input data, for example by identifying some appropriate classifications between groups of data based on common characteristics (clustering). In this way, the model itself generates the possible values of Y and this can be used to make predictions about the new inputs (Figure 11B). 

#### 4.9.2. Regression, Classification, and Clustering Problems

**Figure 12:**
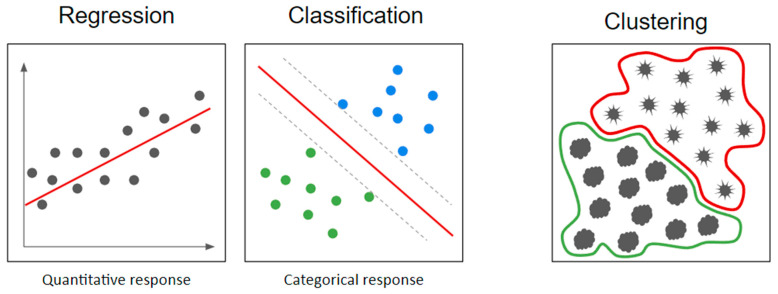
Regression, classification and clustering tasks.

The class of the target variable divides supervised learning problems into two main tasks: problems with a quantitative response are usually referred to as regression problems, and problems involving a qualitative response are often referred to as classification problems (the class being either binary or multi-level) [25,30]. This distinction is key because it guides the choice of the model (or models) to be fitted (Figure 12). Some models are only suitable for a specific task: for example, linear regression is a popular regression technique, while others can be used for both quantitative and qualitative responses [25,30].

Concerning unsupervised learning, the task of main interest in the radiomics field is clustering [79,119]. Clustering models are ML techniques used to group a set of data into homogeneous subgroups called clusters [30]; these models aim to identify structures and patterns within the data, ensuring that elements within a cluster are more similar to each other than to elements in other clusters [30].

Most ML algorithms have one or more adjustable settings, known as hyperparameters, which can be modified to influence the model’s performance. Hyperparameters are parameters external to the model itself that influence its behavior during training, such as the depth of a decision tree or the learning rate of a neural network. In practice, hyperparameter adjustment is shaped by optimizing the model’s performance across multiple subsets of data, often through cross-validation, to find the best configuration that balances accuracy and generalization.

#### 4.9.3. Model Selection

**Figure 13:**
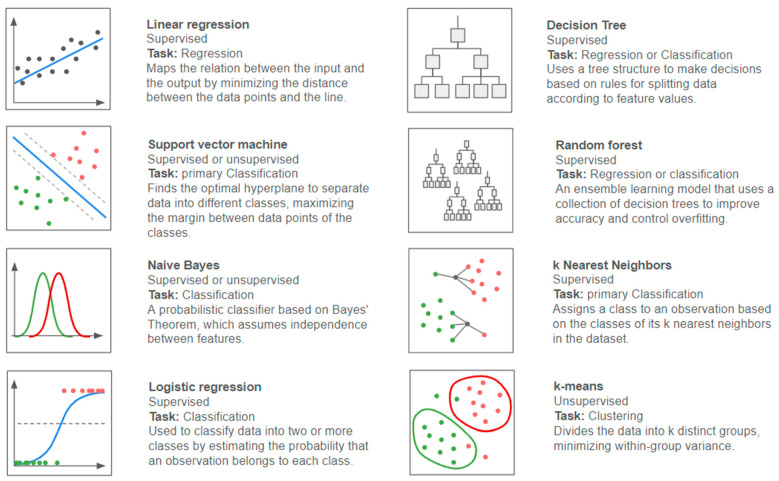
Examples of machine learning models.

**Figure 14:**
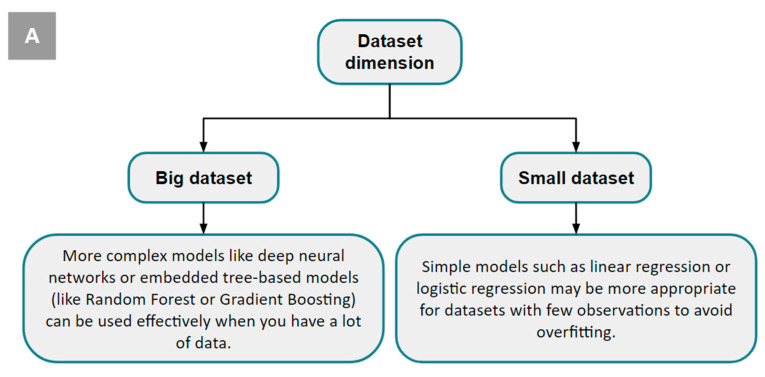

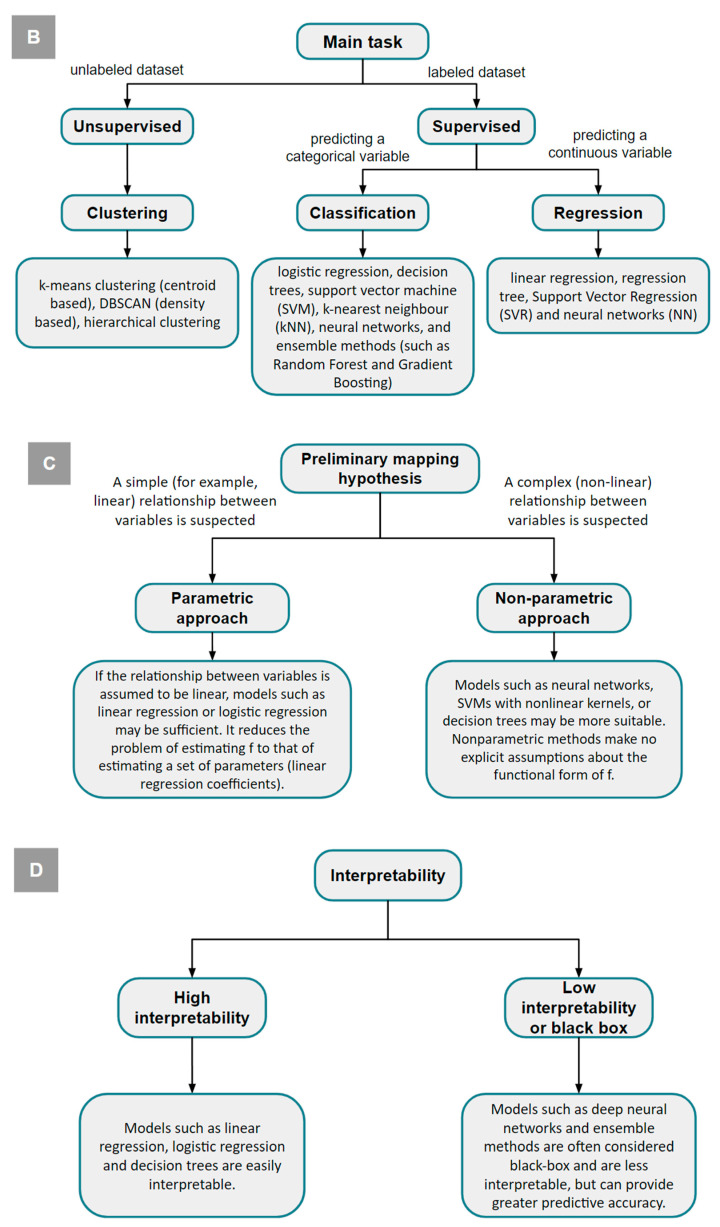
Criteria for model selection. The choice of the best model depends on different factors: the size of the dataset (**A**), the main task that should be executed by the model itself (**B**), the relationship between the variables (**C**), and the interpretability (**D**).

There is no one-size-fits-all ML model (Figure 13). Each algorithm has advantages and disadvantages, and its performance is determined by the type of data used for training and the characteristics of the problem being solved (Figure 14).

#### 4.9.4. Data Partition for Training, Validation, and Test Phases

**Figure 15:**
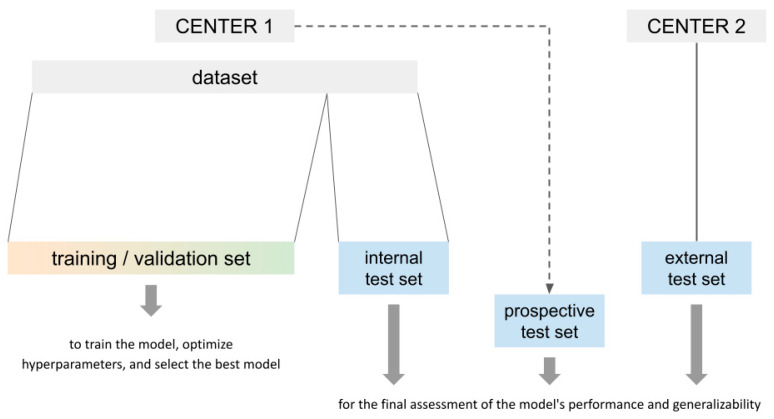
Dataset partition for training, validation, and testing. See also paragraphs 9.4.5 and 9.5.

A standard ML workflow includes training, validation, and testing phases, which require appropriate data partition (Figure 15) [25,30]. In the training phase, the model learns patterns from the data, adjusting its parameters to minimize predictive errors. In the validation phase, the model’s performance is evaluated on a separate dataset, which is used to optimize hyperparameters and prevent overfitting. In radiomics and ML pipelines, the concept of “validation” can often be misunderstood, hence terms like “validation set” and “test set” are used interchangeably in some studies, despite their distinct purposes. The training set is used to develop the model, while the validation set is used to tune parameters and prevent overfitting during model development [14]. In the test phase, the final performance of the model is measured on an independent dataset that was not used during training or validation, to assess its ability to generalize to new data [14,25,30]. 

In radiomics studies, the level at which data are split is a crucial aspect of data preparation and significantly impacts the validity and generalizability of the results. The data can be split at various levels, such as patient-wise, image-wise, study-wise, scanner-wise, or institution-wise, each having distinct implications. In radiomics studies, it is crucial to split data at the patient-level, ensuring that all information for a single patient is contained within the same partition (METRICS item 18 [20]). This approach prevents biases in model integrity and avoids overfitting, which can compromise performance evaluation.

#### 4.9.5. Validation

Validation consists in the process of fine-tuning the model’s architecture by adjusting hyperparameters to prevent overfitting [14,25,30]. It is performed using an independent dataset, distinct from the training set, or through resampling methods, like cross-validation (CV), where the model is run multiple times on different subsets of the training data. This process ensure that the model does not become too closely aligned with any specific patterns in the training data, thereby enhancing its generalizability to unseen data.

**Figure 16:**
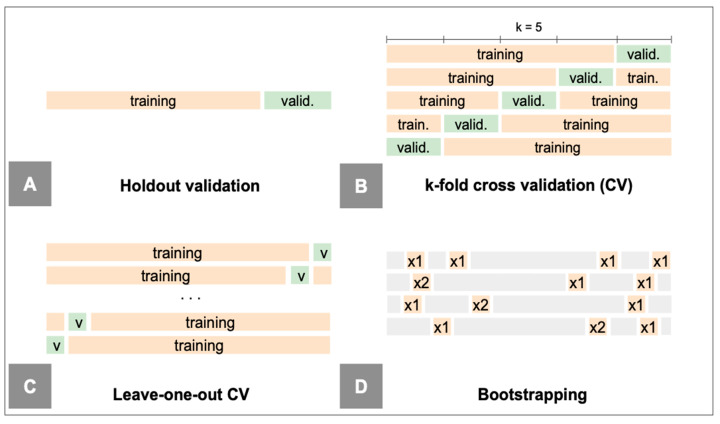
Validation methods. (**A**) Holdout validation. The training/validation set is randomly divided into two parts: a training set and a validation set or hold-out set. The model is fit on the training set, and the fitted model is used to predict the responses for the observations in the validation set. The resulting validation set error rate provides an estimate of the test error rate. The split ratio is usually 70/30 or 80/20. (**B**) Cross-validation. The dataset is divided into k equal-sized subsets (folds, usually k = 5 or 10). The model is trained k times, each time using k-1 folds for training and the remaining fold for validation. The model’s performance is then averaged over the k iterations to obtain a more robust and reliable estimate. Stratified k-fold cross-validation is a variant that preserves the proportion of classes in the folds, particularly useful in cases of class imbalance. (**C**) Leave-one-out cross-validation: an extreme form of cross-validation, where k is equal to the number of observations in the dataset. Each observation is used once as the validation set, while the remaining n-1 observations are used for training. (**D**) Bootstrap. From the original dataset, n observations are randomly selected with replacement to produce B bootstrap datasets (B usually from 100 to 1000). The model is trained on each bootstrap sample and validated on the data not included in that sample (out-of-bag samples). The model’s performance is averaged over the various bootstrap cycles to provide an estimate of its generalization.

Each validation method (Figure 16) has advantages and disadvantages (Table 6) [14,25,30,120]. The simplest approach consists of evaluating the model on a subset of data left aside specifically for this purpose (hold-out dataset). In practice, to avoid sampling issues, resampling methods are commonly preferred: they consist of repeatedly taking samples from a training set and re-fitting a model of interest on each sample in order to obtain additional information about the fitted model.

Once the training and validation datasets are defined, it is important to verify that their feature distributions are similar. This ensures that patterns observed in the training data are likely present in the validation data. Common univariate tests, such as the Mann–Whitney *U*, Kolmogorov–Smirnov, or Shapiro–Wilk tests, are used to compare medians or distributions. These tests are unsupervised, meaning they do not use outcome data and do not violate the rule of reserving the validation set for model testing [14,16].

### 4.10. Model Testing and Performance Metrics

After a model has been trained and validated, testing is a crucial phase to provide a reliable estimate of its performances and generalization capability (METRICS items 26 and 27 [20]) [20,121,122]. 

Typically the test set is a partition of the initial dataset, kept separate and blind from the training and validation data (internal test set). Ideally, to avoid random sampling issues, an external test set acquired from a different center should be used to better assess the model’s performance in a real-world scenario (METRICS 27 [20]). Additionally, it is also possible to acquire new data with a temporal gap after model development; this “prospective test set” allows for an ongoing evaluation of the model’s performance as new data becomes available.

The use of appropriate performance metrics is necessary to correctly evaluate models’ performances (Table 7) (METRICS item 20 [20]) [122].

### 4.11. Model Uncertainty Assessment and Calibration

Uncertainty assessment (METRICS item 21 [20]) and model calibration (METRICS item 22 [20]) are crucial steps for enhancing the reliability and interpretability of ML models, especially in high-stakes fields, like medical imaging and radiomics [20].

The uncertainty assessment involves quantifying the confidence intervals of a model’s predictions, which can be achieved through techniques such as Bayesian inference, bootstrapping, or ensemble methods [25,123,124]. Bootstrapping is one of the most popular techniques and involves running multiple tests by resampling with replacement from the testing dataset [124].

Calibration, on the other hand, focuses on aligning the predicted probabilities with actual outcomes [20,125,126]. A well-calibrated model produces probability estimates that reflect true likelihoods, which is vital for risk assessment and decision-making processes. Calibration techniques, such as Platt scaling or isotonic regression, adjust the model’s output probabilities to better match the observed frequencies [125].

### 4.12. Model Comparison

Many radiomic studies develop several models to proof the added value of a radiomics approach, incorporating different types of predictors, such as clinical predictors only, radiomic features only, or a combination of radiomic and clinical variables. Therefore, model comparison is essential to identify the most effective predictive models and assess the superiority of the radiomics approach to traditional ones.

In multi-parametric studies, such as those involving different MRI sequences, like T2-weighted and diffusion-weighted images, it is important to perform also uni-parametric evaluations to demonstrate the added value and provide a clear justification for adopting a multi-parametric model (METRICS item 23 [20]).

Moreover, studies should compare their methods to non-radiomic approaches commonly used in clinical practice, such as those including semantic features, RADS or RECIST scoring, and simple volume or size evaluations (METRICS item 24 [20]) [39,127]. If non-radiomic methods are not available, proof of improved diagnostic accuracy or patient outcomes, such as improved radiologist performance or overall survival, should be evaluated.

Additionally, studies should include a comparison with a simple baseline reference model, such as a Zero Rules/No Information Rate classifier, to justify the use of more complex ML methods by demonstrating increased performance (METRICS item 25 [20]) [128,129,130].

A comparison of different models should use appropriate statistical methods. The DeLong test is a widely used non-parametric approach to compare the areas under the ROC (receiver operating characteristic) curves of two or more classification models [131,132]. The McNemar’s test is used for paired nominal data to determine whether there are differences in a dichotomous dependent variable between two related groups and is often applied to the confusion matrices directly by evaluating the differences in their misclassification rates [133]. Other methods include decision curve analysis for net benefit comparison, and the Net Reclassification Index [134,135,136]. To address the issue of multiple comparisons, which can inflate the risk of type I errors, multiple testing correction methods, such as Bonferroni, Benjamini–Hochberg, or Tukey, are applied [136].

## 5. Challenges and Future Perspectives: Collaborative Science and Clinical Translatability

In this review, we outlined a comprehensive guide that covers every stage of the machine learning workflow in radiomics studies, with specific references to the METRICS tool [20].

In recent decades, there has been an ongoing debate about the reliability of scientific publications, with concerns that a significant amount of research may be at least inadequately supported by the data and methodologies [137]. Radiomics is not exempt from this scrutiny, and a clear concern is the discrepancy between the large number of published radiomics studies and the limited clinical implementation of these models [138,139]. Consequently, a rigorous evaluation of the methodology in radiomics studies is essential to generate more robust evidence for AI’s efficacy and clinical application (METRICS item 30 [20]). However, it is important to remember that the challenges extend beyond the methodology; economic, organizational, educational, and regulatory issues must also be addressed [6,9,140,141].

In 2017, the Radiomics Quality Score (RQS) was introduced to assess the quality of the radiomics workflow across steps such as image protocol, feature selection, model building, validation, and cost-effectiveness, providing a cumulative percentage score for evaluating radiomics studies [10,14,142]. In 2020, the USA-based CLAIM and MINIMAR checklists were introduced to define the best AI practices, particularly stressing the use of independent datasets for model training and validation. More recently, the METRICS tool was developed through a large international expert panel using a modified Delphi protocol to assess the quality of radiomics research [20]. Guidelines like SPIRIT-AI, CONSORT-AI, and DECIDE-AI offer recommendations for evaluating AI tools in trials [143,144,145].

Some authors believe that the main obstacle to clinical implementation is the lack of model-, code-, and data-sharing practices [138]. Transparency is essential for ensuring reproducibility and comparability across studies, which is a cornerstone of robust scientific research. For this reason, the availability of imaging data (METRICS item 28 [20]) and code (METRICS item 29 [20]) is crucial in radiomics research.

Open code and software enable researchers to replicate studies, independently validate findings, and build upon existing work [146,147]. By making code publicly available, researchers can identify and correct errors, optimize algorithms, and enhance the overall robustness of radiomics models. The introduction of large, open source image repositories offers access to diverse and extensive datasets, addressing the key challenges of radiomics related to reproducibility and standardization [148,149,150]. Recently, RadiomicsHub was proposed as a comprehensive public repository, featuring radiomics data derived from a systematic review of public cancer imaging datasets [151]. The repository includes 29 datasets with segmentations and labels for health outcomes, tumor pathology, and imaging-based scores, encompassing 10,354 patients and 49,515 scans. Of these, 15 datasets are under Creative Commons licenses, while the rest have custom or restricted licensing.

Finally, open datasets also contribute to democratizing research, allowing smaller institutions and less-funded researchers to contribute to and benefit from the collective knowledge base. Ultimately, collaborative science is essential to overcome the need for multidisciplinary expertise in radiomics [131,132,133]. This collective cross-fertilization approach of engineers, physicians, and biostatisticians accelerates the advancement and clinical implementation of radiomics models. The use of cloud-based infrastructures stands as the new frontier for implementing and sharing transparent, reproducible AI-based radiology pipelines, and can further enhanced radiomics research [147]. By providing a consistent computational environment, streamlining data exploration and access, and enabling the storage and sharing of code and results, these platforms support the creation and dissemination of fully reproducible AI pipelines that enhance original research publications [147].

## Figures and Tables

**Table 1:** METRICS items and corresponding paragraphs.

Item/Condition		Paragraph
*Item 1*	Adherence to checklists	4.1 Checklist and guidelines
*Item 2*	Eligibility criteria	4.1.2 Eligibility
*Item 3*	High-quality reference standard	4.1.3 Reference standard
*Item 4*	Multi-centric	4.1.4 Monocentric vs. multicentric
*Item 5*	Standardized imaging protocol	4.1.5 Imaging protocol
*Item 6*	Acquisition parameters	4.1.5 Imaging protocol
*Item 7*	Time interval imaging-ref.std.	4.1.3 Reference standard
*Condition 1*	Segmentation?	4.3 Segmentation
*Condition 2*	Fully automated segmentation?	4.3 Segmentation
*Item 8*	Segmentation method	4.3 Segmentation
*Item 9*	Formal evaluation segm. meth.	4.3 Segmentation
*Item 10*	Test segmentation	4.3 Segmentation
*Condition 3*	Hand-crafted features?	4.4.1 Hand-crafted features
*Item 11*	Image preprocessing	4.2 Image preprocessing
*Item 12*	Standardized feat. extraction soft.	4.4.1 Hand-crafted features
*Item 13*	Extraction parameters	4.4.1 Hand-crafted features
*Condition 4*	Tabular data?	4.5 Tabular data
*Condition 5*	End-to-end deep learning?	1. Introduction, Figure 1
*Item 14*	Removal non-robust features	4.7 Features robustness
*Item 15*	Removal redundant features	4.8 Features selection and regularization
*Item 16*	Dimensionality compared to data size	4.8 Features selection and regularization
*Item 17*	Robustness E2E DL pipeline	4.7 Features robustness
*Item 18*	Data partitioning (train./val./test.)	4.9 Data partition for training, validation, and test
*Item 19*	Confounding factors	4.6 Confounding factors
*Item 20*	Appropriate performance metrics	4.10 Model testing and performance metrics
*Item 21*	Uncertainty assessment	4.11 Model uncertainty assessment and calibration
*Item 22*	Calibration	4.11 Model uncertainty assessment and calibration
*Item 23*	Uni-parametric or proof of added value	4.12 Model comparison
*Item 24*	Comparison with non-radiomics	4.12 Model comparison
*Item 25*	Comparison with classic stat. model	4.12 Model comparison
*Item 26*	Internal testing	4.10 Model testing and performance metrics
*Item 27*	External testing	4.10 Model testing and performance metrics
*Item 28*	Data availability	4.13 Challenges and future perspectives
*Item 29*	Code availability	4.13 Challenges and future perspectives
*Item 30*	Model availability	4.13 Challenges and future perspectives

**Table 2:** Hand-crafted features (adapted from [19]).

*Feature Categories*	*Example Radiomic Features*	*Description*
***First-order***	Mean, median Max/mean/min intensity 10–90th percentile Skewness, Kurtosis Range, Variance Root Mean Squared (RMS) Standard Deviation (SD) Mean Absolut Deviation (MAD) … Area Volume Maximum 3D diameter Major axis length Minor axis length Surface area Elongation Flatness Sphericity	First-order features include basic statistics on the distribution of the values of individual voxels, disregarding spatial relationship, or shape-based features.
***Second-order***	Gray level co-occurrence matrix Gray-level run length matrix Gray-level size zone matrix Neighboring Gray Tone Difference Matrix Gray Levele Dependence Matrix	Second-order features describe the statistical relationships between pixels or voxels.
***High-order***	Autoregressive model Haar wavelet	High-order features are usually based on matrices that consider relationships between three or more pixels or voxels.

**Table 3:** Intraclass Correlation Coefficient (ICC) and the Concordance Correlation Coefficient (CCC).

*Subset Search Process*	*What It Represents*	*When It Can Be Used*
*Intraclass Correlation Coefficient (ICC)*	The ICC is useful for evaluating the reproducibility or reliability of measurements between different repetitions or between different assessments made by different observers. A high ICC indicates that most variability is due to genuine differences between subjects, suggesting feature robustness.	If a test–retest is performed (the same image from the same patient and scanner obtained a few minutes apart), then the ICC can be calculated.
*Concordance Correlation Coefficient (CCC)*	It combines measures of precision and accuracy to assess how well bivariate pairs of observations conform relative to a gold standard or another set. It is valuable for comparing the agreement between features extracted with different imaging techniques or acquisition parameters.	If multiple phantom images for the same and different scanners can be acquired, then the CCC for each feature can be calculated.

**Table 4:** Statistical-based filtering methods.

*Statistical Method*	*Exclusion Criteria*
*Missing Percentage*	Disproportionate share of missing samples and difficult to fill
*Variance*	Variance close to or equal to 0
*Frequency*	Features excessively concentrated in one category of values
*Correlation Coefficients (Spearman, Pearson, and Kendall)*	Correlation coefficients close to or equal to 0
*Analysis of Variance (ANOVA)*	Too-low *F*-value or excluded features with a *p*-value < 0.05
*χ^2^ Test*	Too-low χ^2^ value or *p*-value < 0.05
*Mutual Information*	Mutual information close to or equal to 0
*mRMR (Minimum Redundancy Maximum Relevance)*	Features with the minimum correlation and maximum redundancy
*Fisher Score*	Large intraclass distances and small interclass distances

**Table 5:** Wrapper methods.

*Subset Search Process*	*Subset Search Method*	*Criteria*
*Complete search*	Breadth First Search Best First Search	Iterate through all possible combinations of feature subsets, then select the feature subset with the best model score. High computational cost
*Heuristic search*	Sequential Forward Selection Sequential Backward Selection Bidirectional Search Plus-L Minus-R Selection Sequential Floating Selection Decision Tree Method	Uses rules or guided search strategies to find a good subset of features, without necessarily guaranteeing the optimal solution
*Random search*	Random Generation plus Sequential Selection Simulated Annealing Genetic	A random subset of features is generated and then these feature subsets are evaluated; does not guarantee optimality or computational efficiency

**Table 6:** Comparison of different validation methods.

*Validation Methods*	*Advantages*	*Disadvantages*
*Holdout dataset*	Computationally feasible with respect to LOOCV Lower variance with respect to LOOCV	Higher bias with respect to LOOCV
*Cross-validation (CV)*	Computationally feasible with respect to LOOCV Lower variance with respect to LOOCV	Higher bias with respect to LOOCV
*Leave-one-out CV (LOOCV)*	Useful for a small dataset Lower bias compared to CV	Computationally intensive Lower variance compared to CV
*Bootstrapping*	Can be applied in almost all situations Makes the most of available data without the need for a separate validation set Can also be used to quantify the uncertainty associated with a given estimator or statistical learning method (i.e., to calculate the confidence intervals)	Can lead to an overestimation of the model’s performance

**Table 7:** Main performance metrics and explanations.

*Performance Metrics*		*Meaning*	*Description*
*Regression task*	Residual standard error (RSE)	It represents the standard deviation of the residuals, which are the differences between the observed values and the values predicted by the model. It provides an estimate of the typical size of the prediction errors.	It is calculated as the square root of the sum of squared residuals divided by the degrees of freedom (n − p − 1), where n is the number of observations and p is the number of predictors.
	R^2^ statistic	It represents the proportion of the variance in the dependent variable that is predictable from the independent variables. R^2^ values range from 0 to 1, with 0 indicating that the model does not explain any of the variability in the response data around its mean and 1 indicating that the model explains all the variability.	It is calculated as 1 minus the ratio of the sum of squared residuals to the total sum of squares.
	F-statistic	It compares a model with no predictors (intercept only) to the model being evaluated. A higher F-statistic indicates that the model provides a better fit to the data than a model without any predictors.	It is calculated as the ratio of the mean regression sum of squares to the mean error sum of squares, and its significance is evaluated using the F-distribution.
*Classification task*	Sensitivity	Sensitivity, or true-positive rate, is the ability of a test to correctly identify positive cases.	It is calculated as the ratio of true positives to the sum of true positives and false negatives (TP/(TP + FN))
	Specificity	Specificity, or true-negative rate, is the ability of a test to correctly identify negative cases.	It is calculated as the ratio of true negatives to the sum of true negatives and false positives (TN/(TN + FP)).
	Accuracy	Accuracy is the overall correctness of a test.	It is calculated as the ratio of the number of correct predictions (true positives and true negatives) to the total number of cases examined ((TP + TN)/(TP + TN + FP + FN)).
	Precision	Precision, or positive predictive value, indicates the proportion of correct positive results among all those identified as positive.	It is calculated as the ratio of true positives to the sum of true positives and false positives (TP/(TP + FP)).
	Recall	Recall is synonymous with sensitivity.	
	F1 score	The F1 score is the harmonic mean of precision and recall, providing a single measure that balances both aspects.	It is calculated as 2 × (Precision × Recall)/(Precision + Recall).
	Receiver operating characteristic (ROC) curves and area under the curve (AUC)	Depict the trade-off between the true-positive rate (sensitivity) and the false-positive rate (1-specificity) across various classification thresholds. A higher area under the ROC curve signifies a better discrimination capability, with an AUC of 1 indicating a perfect classifier.	
	Confusion matrices	They present the counts of true-positive, true-negative, false-positive, and false-negative predictions, providing insights into the model’s ability to correctly classify instances from each class. From the confusion matrix, derived metrics, such as accuracy, precision, recall (sensitivity), specificity, and F1-score, can be calculated.	
