# Correction: Cè et al. Decoding Radiomics: A Step-by-Step Guide to Machine Learning Workflow in Hand-Crafted and Deep Learning Radiomics Studies. *Diagnostics* 2024, *14*, 2473

**DOI:** 10.3390/diagnostics16091301

**Published:** 2026-04-27

**Authors:** Maurizio Cè, Marius Dumitru Chiriac, Andrea Cozzi, Laura Macrì, Francesca Lucrezia Rabaiotti, Giovanni Irmici, Deborah Fazzini, Gianpaolo Carrafiello, Michaela Cellina

**Affiliations:** 1 Postgraduation School in Radiodiagnostics, Università degli Studi di Milano, Via Festa del Perdono 7, 20122 Milan, Italy; 2 Politecnico di Milano, Piazza Leonardo da Vinci 32, 20133 Milan, Italy; 3 Imaging Institute of Southern Switzerland (IIMSI), Ente Ospedaliero Cantonale (EOC), Via Tesserete 46, 6900 Lugano, Switzerland; 4 Breast Imaging Department, Fondazione IRCCS Istituto Nazionale dei Tumori, Via Giacomo Venezian 1, 20133 Milan, Italy; 5 Radiology Department, Centro Diagnostico Italiano, Via Saint Bon 20, 20147 Milan, Italy; 6 Radiology Department, Fondazione IRCCS Cà Granda Ospedale Maggiore Policlinico, Via Francesco Sforza 35, 20122 Milan, Italy; 7 Department of Oncology and Hematology-Oncology, Università degli Studi di Milano, Via Festa del Perdono 7, 20122 Milan, Italy; 8 Radiology Department, ASST Fatebenefratelli Sacco, Piazza Principessa Clotilde 3, 20121 Milan, Italy

## 1. Error in Figure

In the original publication [1], Figure 2 was inaccurate, as suggested by a reader on PubPeer. The tip of the arrow was therefore moved towards “modeling” and an asterisk was added to stress the concept that feature extraction and processing should be performed solely on the training set after data partitioning, as clearly stated in the text. The new version of Figure 2 and the figure legend appear below.

In the original publication [1], there was a typo in Figure 4: the word “high” was duplicated. The new version of Figure 4 and the figure legend appear below.

## 2. Text Correction

The second-to-last paragraph of Section 3 was amended as follows: “By introducing regularization and feature selection, the model’s variance ***reduces*** and the model’s performance on unseen data improves, at the cost of slightly increasing the model’s bias. Underfitting occurs when a model has high bias and ***low*** variance, resulting in a poor performance for both training and testing data. This often happens when the model is too simple or the training data are insufficient.”

The first sentence of Section 4.8.1 was updated as follows: “Not all extracted features are useful for modeling, hence the need for feature selection (METRICS item 15 [20]) [111], which must be performed solely on the training set.”

The authors state that the scientific conclusions are unaffected. This correction was approved by the Academic Editor. The original publication has also been updated.

## Figures and Tables

**Figure 2:**
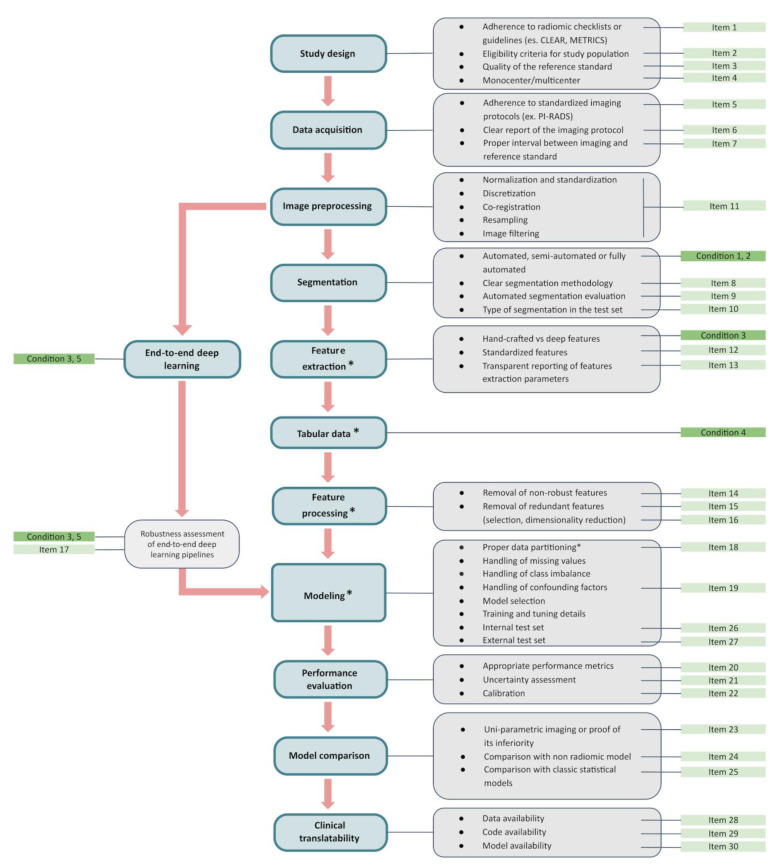
The main steps, the specific sub-tasks for each phase, and the related METRICS items. * To avoid data leakage and overfitting, data partitioning should be performed before feature extraction, processing, and model training.

**Figure 4:**
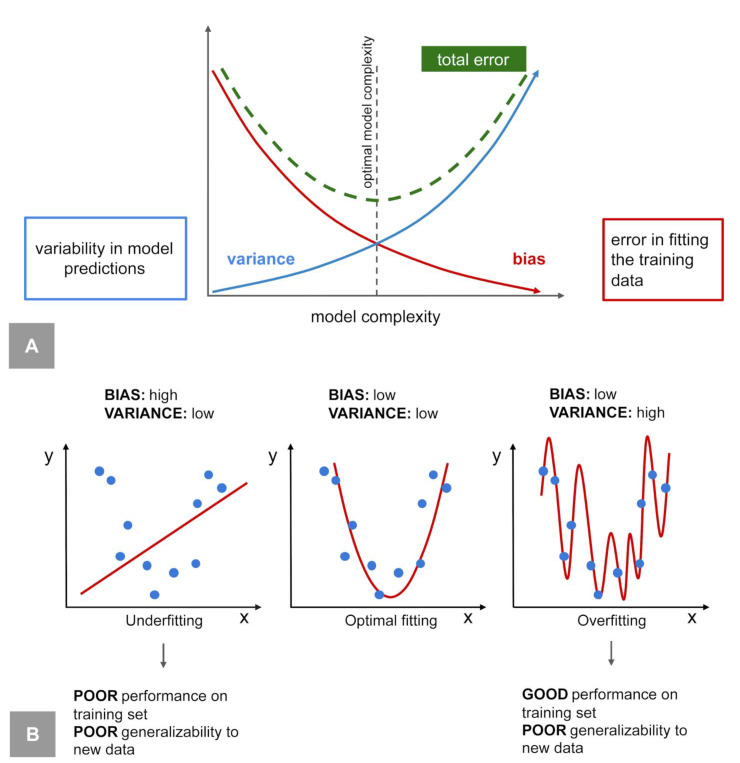
Bias–variance trade-off, overfitting, and underfitting. (**A**) Bias–variance trade-off. As the complexity of the model increases, the bias decreases but the variance increases. More complex models can capture intricate patterns in the data, better fitting the training dataset and reducing systematic errors (bias), but they also become more sensitive to noise and specific data points, leading to higher variability in model predictions (variance). (**B**) Overfitting and underfitting. Overfitting occurs when a model captures noise and fluctuations in the training data rather than the underlying patterns, resulting in an excellent performance on the training set (low bias) but poor generalizability to new data (high variance). Underfitting happens when a model is too simple to detect the underlying patterns, leading to a poor performance on both the training (high bias) and testing datasets.
